# Perceptions of strategies to facilitate caring for patients in primary health care clinics

**DOI:** 10.4102/phcfm.v13i1.2652

**Published:** 2021-02-18

**Authors:** Tintswalo V. Nesengani, Charlene Downing, Marie Poggenpoel, Chris Stein

**Affiliations:** 1 Department of Nursing, Faculty of Health Science, University of Johannesburg, Johannesburg, South Africa; 2 Department of Emergency Medicine, Faculty of Health Sciences, University of Johannesburg, Johannesburg, South Africa

**Keywords:** effective caring, facilitate, PHC clinic, PHC professional nurse, strategies

## Abstract

**Background:**

Caring in nursing helps patients feel better, whilst the absence of caring will affect patients psychologically, emotionally and physically.

**Aim:**

The aim of this article was to explore and describe primary health care (PHC) professional nurses’ and PHC nurse managers’ perceptions of the developed strategies to facilitate effective caring for patients in PHC clinics.

**Setting:**

This study was conducted in two PHC clinics in Ekurhuleni, an area east of the Gauteng province, South Africa.

**Methods:**

The study used a qualitative, exploratory, descriptive and contextual design. In-depth individual phenomenological interviews were conducted with eight purposively selected PHC professional nurses working in PHC clinics and two PHC nurse managers supervising PHC clinics in Ekurhuleni. Giorgi’s coding method was used to analyse the data.

**Results:**

Three themes were identified from the results of the exploration and description of PHC professional nurses’ and PHC nurse managers’ perceptions of the developed strategies. The use of active listening skills, showing interest in what is being said, asking questions and providing constructive feedback that focuses on the issue were the most effective strategies in improving effective communication between PHC nurse managers and PHC professional nurses. The PHC professional nurses were encouraged to put patients’ interests first whilst adhering to the ethical principles of nursing.

**Conclusion:**

Although caring is considered as the core of nursing practice, PHC professional nurses and PHC nurse managers perceive that rendering effective caring for patients needs to be reinforced through the use of strategies that will enable change and improve clinical practice in PHC clinics.

## Introduction

Caring is the core concept in nursing and includes care for and care about clients. Care for, being the first of the two main domains in holistic nursing, is related to professional knowledge and expertise, whilst the second domain relates to the psychological and spiritual consideration of clients.^1^ Nursing is a caring profession that should be coupled with the provision of excellent care within an ethical, reflective and knowing framework.^2^ In general, caring actions by professional nurses are basically known to relate to helping patients to alleviate their pain and distress in a systematic way. Whilst also associated with qualities of respect, patience, trust and honesty, effective communication, dedication and a positive attitude are essential qualities in effective caring for patients.^2^ To ensure caring in nursing, the caring actions should help patients feel better, whilst the absence of caring would affect patients psychologically, emotionally and physically.^2,3^ In South Africa, clinics have become the cornerstone of the public health system. In this regard, it is necessary and expected that clinics provide comprehensive and integrated basic health programmes, with members of the public being treated with care, respect and compassion by all healthcare professionals.^4^ Primary healthcare (PHC) clinics^5^ are regarded as the point of entry for patients in need of preventative care and diagnosis and treatment of minor ailments. In this study, PHC professional nurses, referred to as primary care specialists,^6^ are responsible for the provision of direct care to patients with all types of illnesses and ailments, offering the first level of nursing care that patients receive in clinics.

In a qualitative research study by Nesengani,^7^ eight in-depth individual phenomenological interviews were conducted with eight purposively selected PHC professional nurses regarding their experiences of caring for patients in PHC clinics. The findings of this study reflected three themes: empowering experiences in caring for patients, disempowering experiences of caring for patients and the negative experiences with the PHC clinic systems. The empowering experiences of caring for patients included effective caring for patients, appreciative behaviours by patients and constructive management practices. Disempowering experiences in caring for patients referred to negative experiences with colleagues, negative experiences with patients and negative experiences with management practices. Lastly, negative experiences with PHC clinic systems included shortage of resources, referring to shortage of medicines; functional medical equipment and PHC professional nurses; high workload of PHC professional nurses; low salaries; no rewards and high resignations by PHC professional nurses; and unavailable ambulances.^7^

Various studies on experiences of professional nurses in PHC clinical settings were also conducted in different parts of the world, and findings similar to a research study conducted by Nesengani^7^ were reported. In a research study entitled ‘Primary health care: The experiences of nurses’ conducted in Chile, Garcίa-Vera et al.^8^ reported that even though the professional nurses were grateful of their work, they had negative experiences. This included experiences of work overload, which was revealed as professional nurses having many patients waiting to be seen. This was expressed as one of the experiences which made these professional nurses to feel pressured, very tired and overwhelmed by the number of patients and paperwork. The findings from this study also revealed that these professional nurses felt that the system they were part of was wearing them down mentally more than physically.

In another study conducted by Mayers,^9^ which explored the experiences of nurses in PHC in South Africa, it was revealed that the challenges in the provision of primary healthcare in the public sector included long waiting times, excessive workloads for staff, poor attitudes, rudeness and favouritism of nurses, accompanied by a lack of confidentiality – particularly in large urban clinics. Other factors such as an inadequate distribution of resources, inadequate or irregular supply of basic medicines and the inefficient interactions amongst different hierarchical levels of public health services were also mentioned, which were considered as factors that further compromised the services provided.

Based on the findings of the qualitative research study by Nesengani,^7^ the researcher developed and implemented strategies to facilitate effective caring for patients in PHC clinics in Ekurhuleni, which was Phase 1 of the study. After the implementation of the developed strategies for a period of 12 months in some PHC clinics, improvement was observed in the delivery of healthcare services by PHC professional nurses through showing of commitment, respect and willingness to help those patients in need.^7^ In this study, which is Phase 2 of the study, the researcher explored and described PHC professional nurses’ and PHC nurse managers’ perceptions regarding the developed strategies to facilitate effective caring for patients in PHC clinics. Exploring and describing the participants’ perceptions of the developed strategies in this study did not only provide information about the practical effectiveness of the developed strategies but also provided information on the participants’ views on those aspects that they regarded as most important. The study also provided information on the participants’ views on the need to implement and maintain the strategies in all PHC clinics in Ekurhuleni.

## Methods

### Study design

A qualitative, exploratory, descriptive and contextual research study design^10,11,12^ was used to explore and describe PHC professional nurses’ and PHC nurse managers’ perceptions of the developed strategies to facilitate effective caring for patients in PHC clinics in Ekurhuleni. The study took place in two phases. in Phase 1, the findings of a previously conducted research study^7^ were utilised as a basis for developing strategies. In Phase 2, the study explored and described PHC professional nurses’ and PHC nurse managers’ perceptions of the developed strategies. This article only reports on the findings of Phase 2 of the study.

### Setting

The setting of this study was the PHC clinics in Ekurhuleni, an area in the eastern part of Gauteng province, South Africa. Of the nine provinces of South Africa, Gauteng is the smallest in terms of land area but overpopulated. Gauteng province encompasses about 15.5 million people of diverse cultures, languages and belief practices.^13^ The name ‘Ekurhuleni’ is derived from the Xitsonga language, which means ‘place of peace.’ Ekurhuleni is one of the most densely populated areas in the province and the country. It has 94 public health clinics and seven public hospitals, which render healthcare services to its community. The PHC clinics provide comprehensive healthcare services through the district health system, which is part of the provincial healthcare system.^4^

### Population and sampling

The sampling frame showing a list of clinics from which to draw the sample^14^ was developed. Two PHC clinics, both community health centres that were similar in terms of healthcare services rendered to patients, had a patient headcount of above 5000 per month and readily accessible to the researcher, were sampled and used as research sites in the eastern part of Gauteng province. The rationale for choosing these healthcare facilities is that the two PHC clinics experienced similar challenges of being under-resourced in terms of available staff with relevant qualifications and experience to work in PHC clinics. Other challenges experienced by the sampled PHC clinics were the high number of patients that the PHC professional nurses had to deal with on a daily basis, long waiting times for patients and the inadequate or irregular supply of basic medicines, resulting in compromised patient care.

The study population comprised professional nurses and nurse managers in the PHC clinics in Gauteng province. The sample consisted of eight PHC professional nurses and two PHC nurse managers, purposively selected as guided by the purpose of the study.^15,16^ The inclusion criteria for participants were as follows: professional nurses working in PHC clinics in Gauteng province for 2 years or more and expressing willingness to share information with the researcher. The criteria for PHC nurse managers’ inclusion in the study were as follows: those with 2 years or more experience, supervising PHC clinics in Gauteng province and expressing willingness to share information with the researcher. The rationale for choosing the two categories of participants was to include those who had the relevant lived experience and could give a good perspective with regard to their perceptions of the developed strategies to facilitate effective caring for patients, and also to include those who had sufficient knowledge in the PHC clinics and could account for the caring rendered to patients in the same environment. The sampled participants were considered information-rich^16^ because they were seen as individuals who were most likely to provide data of sufficient relevance and depth. Data collection was performed until data saturation was reached.^16^ To ensure transferability, the researcher gave a rich description of the findings in such a manner that another person can compare it with the findings of other studies.^12^ Findings from the participants added value or significance to the study because their views and opinions were drawn from their perceptions of the developed strategies.

### Data collection

Data were collected by means of in-depth, individual phenomenological interviews. The focus of the interviews was to explore and describe participants’ perceptions of the developed strategies,^17^ implemented in some PHC clinics in Ekurhuleni for a period of 12 months (January 2018–January 2019). The researcher considered exploration and description^18^ of the strategies necessary in order to determine the views, significance and the practical effectiveness of the strategies. The interviews were conducted by the researcher between January and February 2019, on Friday afternoons, when PHC clinics were not busy. Each interview lasted for approximately 45–60 min and was held in quiet consulting rooms away from the patients’ waiting rooms. The consulting rooms ensured no interruption, privacy of the participants and enabled the researcher and participants to concentrate and engage more on the interview process. The researcher used a high-quality audiotape^19^ to record all interviews, which were transcribed verbatim during data analysis.

The researcher posed the following broad open-ended question in each interview: ‘kindly share with me: what are your perceptions regarding the developed strategies to facilitate effective caring for patients in PHC clinics?.’ This broad open-ended question posed to participants during interviews allowed them to share their views relatively unconstrained by the researcher’s perspective or past research findings.^12^ During the interview process, the researcher used probes under the main question to encourage elaboration and elicit more information.^12^ The researcher used questions such as ‘Anything else?’, ‘And then what happened?’, as well as ‘Tell me more about that’ The researcher avoided leading questions that suggested particular answers, or which framed the participants’ replies to them. The researcher did not interrupt participants but used prompts, which included noises to encourage participants to continue, such as ‘uh-huh’ and ‘mm’, as well as non-verbal cues such as head nodding.^12^ The researcher summarised participants’ last statements to capture the main points of the interview. Member-checking ^17^ was conducted on the spot during the course of the interviews and at the end of each interview. The analysed and interpreted data were sent back to participants in order for them to validate the interpretation made by the researcher, and to suggest changes if they are unhappy with or they had been misrepresented.^17^

### Data analysis

The researcher transcribed the audio-recorded interviews word for word, which was done as soon as^14^ possible after the interview. After the interviews were transcribed, the researcher listened to the recordings, read through the interview transcripts and made notations or observations on them. The initial reading resulted in tentative ideas about categories and relationships in data, which is described by Giorgi as the ‘marking of what is of interest in the text.^14^ Giorgi’s method of thematic analysis was utilised where the researcher identified a list of themes, which served as the initial set of codes by going through the whole transcript. The researcher derived themes by looking for topics that came up frequently and looking for similarities.

### Measures to ensure trustworthiness

Measures to ensure trustworthiness were observed throughout the study by using Guba’s model.^20^ According to Krefting,^20^ trustworthiness is a measure of quality of the research, which entails credibility, transferability, dependability and confirmability. The researcher ensured credibility of the research through immersion in the field and by using a variety of strategies to collect data, which included interviews, observation, field notes and reflexive journal.^16,20,21^ The researcher presented the strategies and findings at research forums, and corrections were made based on the recommendations by experts in research. The researcher ensured transferability by providing a rich description of the demographics of participants and description of the findings in such a manner that another person can compare it with the findings of other studies.^12,22^ Dependability was ensured by providing a dense description of the research methodology, supported by literature review. Confirmability is a criterion for trustworthiness in a qualitative inquiry, which refers to the neutrality of the data and interpretations.^16^ To ensure confirmability, the entire research process was audited by the study supervisors and an independent coder who worked closely with the researcher.

## Ethical considerations

In order to ensure ethical conduct by the researcher, the following ethical principles as set out by Dhai and McQuoid-Mason^23^ were adhered to throughout the research process: the principle of respect for autonomy, the principle of non-maleficence, the principle of beneficence and the principle of justice. The researcher obtained ethical clearance from the University of Johannesburg Research Ethics Committee (clearance number: REC-01-160-2016) and the Health District Ethics Committee (clearance number: 30/11/2016-3). Permission was also obtained from the nurse managers of the PHC clinics where the study took place. The researcher gave participants adequate information about the research, ensured that participants comprehended that information and had the ability to voluntarily consent or decline participation.^23^ Confidentiality was ensured by affording participants a promise of confidentiality, which was a pledge that any information participants provided would not be publicly reported in a manner that identifies them.^21^

## Findings

In this study, only findings of Phase 2, the exploration and description of perceptions on the developed strategies, will be reported.

## Demographics of participants

The demographics and characteristics of the participants are presented in Table 1.

**TABLE 1:** Demographics of participants.

Demographic information	Total
**Gender**	
Male	0
Female	10
**Age (years)**	
40–45	2
46–50	4
51–55	4
**Race**	
Black	9
White	1
Mixed race	0
Other	0
**Experience (years)**	
5–10	1
11–15	4
> 20	5
**Educational level**	
Diploma in Clinical Nursing Sciences	10
Diploma in Clinical Assessment, Diagnosis, Treatment and Care	10
Degree in Nursing Sciences	3
**Types of participants**	
PHC professional nurses	8
PHC nurse managers	2

*Source*: Nesengani TV. Strategies for professional nurses to facilitate caring of patients in the Primary Health Care clinics in Gauteng Province, South Africa [PhD thesis]. Johannesburg: University of Johannesburg; 2019.

Based on the feedback provided by participants regarding their perceptions of the developed strategies, three themes and 11 categories were identified during data analysis, which are discussed below. Direct quotations are provided to illustrate participants’ perceptions.

### Theme 1: Perceptions of strategies that focused on improving the healthcare system

In Theme 1, participants’ perceptions of strategies that focused on improving the healthcare system were identified. Under this theme, four categories emerged, namely, strengthening working relationships between PHC professional nurses and the PHC nurse manager; improve and manage PHC professional nurses’ attitudes towards the PHC nurse manager, patients and work; Wellness clinics for PHC professional nurses; and improvement in how PHC professional nurses perform duties and engage with each other.

#### Strengthening working relationships between PHC professional nurses and PHC nurse managers

Participants expressed their perceptions regarding the value of the strategies for strengthening working relationships between PHC professional nurses and the PHC nurse managers. This was supported by the following quotations:

‘The strengthening of working relationships is a very good strategy which can also assist our managers to give us continuous feedback, on the spot training, effective management of meetings and to use the line of communication.’ (#PN4, 49 years old, 27 years’ experience)

‘This strategy will help us as managers to learn to use our listening skills effectively, be good observers and act professionally.’ (#NM1, 54 years old, 35 years’ experience)

#### Improve and manage PHC professional nurses’ attitudes towards PHC nurse managers, patients and work

Participants expressed their perceptions of the need to maintain the strategies on improving and managing PHC professional nurses’ attitudes. This was supported by the following quotations:

‘This will improve and manage nurses’ attitudes towards the manager and patients.’ (#PN1, 46 years old, 12 years’ experience)

‘As managers responsible for clinics, l think these strategies will assist us to correct the bad attitudes that our nurses have. I have tried to use the policies of our institution regarding human resource management through the support of these strategies.’ (#NM2, 52 years old, 29 years’ experience)

#### Wellness clinics for PHC professional nurses

In this category, some participants expressed their perceptions of the strategy that deals with providing help for PHC professional nurses, specifically on attending of Wellness clinics to assist them in dealing with their various disempowering experiences, as illustrated by the following quotation:

‘I think this strategy is more relevant to our situation because if PHC nurses can attend a Wellness Clinic, it will ease them from feeling a heavy burden and being impatient towards patients and other staff members.’ (#PN1, 46 years old, 12 years’ experience)

#### Improvement in how PHC professional nurses perform duties and engage with each other

Some participants indicated their perceptions of having seen some improvements in how they perform their duties and how they engage with each other since they started using the strategies in their workplace. This was illustrated by the following quotations:

‘Since we started using this strategy a few months ago in our clinic, l saw a bit of improvement in how we work, even how our manager communicates with us. I think this can be a good strategy to use if followed properly.’ (#PN2, 49 years old, 15 years’ experience)

‘Since we started with these strategies, l can tell you, the nurses are trying to do their bit, our manager is visible in the clinic, everyone is communicating well with each other.’ (#PN3, 52 years old, 30 years’ experience)

### Theme 2: Perceptions of strategies that address essential functions of PHC professional nurses

Theme 2 focused on perceptions of strategies that address essential functions of PHC professional nurses, and two categories were identified: *Back to basics – Batho Pele principles, Patients’ Rights Charter and reinforcement of nursing ethics.*

#### Back to basics – Batho Pele principles, Patients’ Rights’ Charter

The category ‘Back to basics – Batho Pele principles and Patients’ Rights Charter emerged from participants’ feedback, which they revealed to be essential in PHC professional nurses’ effective caring for patients. This was supported by the following quotations:

’The strategies are good for our clinics as the managers will be able to correct bad attitudes by reinforcing Batho Pele Principles amongst the nurses.’ (#PN4, 49 years old, 27 years’ experience)

‘This will help us to go back to the basics of our profession as our minister always says.’ (#PN5, 45 years old, 13 years’ experience)

#### Reinforcement of nursing ethics

Participants gave a positive feedback on the strategies to reinforce nursing ethics, with their perception being that the strategy will remind and guide them in effectively caring for patients, as illustrated by the following quotations:

‘Since we started using these strategies, we have also established an ethics committee in the clinics currently, and it will be able to oversee that we always abide to the professional ethics at all times.’ (#PN4, 49 years old, 27 years’ experience)

‘The application of all the strategies will help us in the reinforcement of nursing ethics.’ (#PN7, 43 years old, 12 years’ experience)

### Theme 3: Perceptions of strategies that address PHC professional nurses’ challenges

The third theme identified the perceptions of strategies that address PHC professional nurses’ challenges, which were considered to have a negative effect on the organisation and on the quality of services rendered to patients. The five categories identified were absenteeism; problems of patients from other countries; shortage of PHC professional nurses; lack of resources – medicines, functional medical equipment and ambulances; poor nurse–patient relationships.

#### Absenteeism

This category is based on the participants’ comments on their perceptions of the strategy to deal with PHC professional nurses’ absenteeism in the workplace. Participants expressed the need to maintain this strategy as absenteeism had a negative effect in terms of the quality of care rendered to patients, as illustrated below:

‘I was part of the group, l won’t say delinquent, but something like that because most of the time l would be absent for no reason. I feel, since we started with the strategies, this strategy helped me a lot, and I think it will also help others to follow the rules of coming to work as required, and not do as I used to do.’ (#PN8, 50 years old, 7 years’ experience)

‘Absenteeism is also rife among our nurses. I will use the strategies to remind our nurses to be professional. We will also keep on reminding them about the relevant organisational policies on absenteeism.’ (#NM2, 52 years old, 29 years’ experience)

#### Problems with patients from other countries

Participants expressed their perceptions of the need to treat patients equally without any discrimination; hence, maintaining the strategies will encourage them to understand patients from other countries, as shown in the following quotations:

‘This will encourage us to understand patients from other nationalities or outside of our country, hence to treat them equally. It will remind us of humanity and to treat all patients without discrimination.’ (#PN3, 52 years old, 30 years’ experience)

‘These strategies encourage us as professional nurses to even treat foreign patients with respect and dignity.’ (#PN6, 52 years old, 32 years’ experience)

#### Shortage of PHC professional nurses

Participants revealed their perceptions that they think that if management can maintain the strategy that deals with addressing the shortage of PHC professional nurses in the PHC clinics, it will improve the services rendered. The following quotations verify the findings:

‘We are few in the clinics as professional nurses but if you look at what we’re doing in terms of all responsibilities, it’s too much for us. We will really appreciate if something can be done about this. I hope with these strategies, our managers will start doing something in order to improve the care we render to patients.’ (#PN6, 52 years old, 32 years’ experience)

‘With the strategy to deal with shortage of nurses, l think this will help resolve the problem we’re having in the clinics. With the number of nurses we are having in clinics, we feel pressured to do more with the few staff members available. I think this can help resolve our challenges.’ (#NM2, 52 years old, 29 years’ experience)

#### Lack of resources: Medicines, functional medical equipment and ambulances

This category is based on the participants’ feedback on their perceptions of the strategy that deals with the lack of resources, such as medicines, functional medical equipment and ambulances. Participants expressed the need to present the strategies to senior management, as this will motivate them to provide basic equipment in the PHC clinics. The following quotations verify the findings:

‘Our hands are tied as we don’t have the resources, budget has been cut. Every now and then when you request for resources, you will be told that there is no money to buy the resources. I feel if these strategies can be presented to those up there, something will be done.’ (#PN5, 45 years old, 13 years’ experience)

‘Shortage of medicines and the basic equipment becomes a problem to all of us if we do not have such things. I would recommend that these strategies be presented to our senior managers in the corporate office because sometimes they see us not performing, meanwhile it’s because of such problems. I think something can be done after seeing these strategies.’ (#NM2, 52 years old, 29 years’ experience)

#### Poor nurse–patient relationships

This category is based on the participants’ feedback on their perceptions of the strategy that deals with managing poor relationships between PHC professional nurses and patients. Participants gave a positive feedback that the strategy is good and they would like to motivate for the strategy to be maintained in order to improve the nurse–patient relationships in the PHC clinics, as illustrated by the following quotations:

‘I think this strategy is good and will address the poor nurse–patient relationships. Currently I have seen an improvement, most of the nurses in the clinic are now trying to communicate well with patients.’ (#PN2, 49 years old, 15 years’ experience)

‘I used to be unfriendly to patients, but now, I can confess that I was wrong and I won’t do it anymore. The strategy assisted me to see the value of our patients as human beings, otherwise previously, I didn’t care.’ (#PN8, 50 years old, 7 years’ experience)

## Discussion

The present study explored and described PHC professional nurses’ and PHC nurse managers’ perceptions of strategies to facilitate effective caring for patients in PHC clinics. Strategies that focused on improving the healthcare system were perceived to be important by participants as they focus on strengthening working relationships between PHC professional nurses and PHC nurse managers. According to Thulth et al.,^24^ the primary factor in professional nurses’ satisfaction and loyalty to the workplace is their relationship with their immediate supervisor and the concern over their satisfaction. The patterns of social connections between individuals, and individuals and groups are described by the structural social capital, which encourages the development of shared meanings and trusting relationships.^25^ Communication between nurses and co-workers, in conjunction with good, interpersonal relationships and social interaction, is considered indispensable conditions for feeling comfortable with one’s work.^26^

Participants in the present study expressed their perceptions that maintaining the strategies for improving and managing PHC professional nurses’ negative attitudes will lead to positive repercussions for the patients and all professionals. Worker performance affects the success of an institution, whilst institutional factors, in turn, affect the performance of workers. It therefore becomes important for the workplace to have a robust institutional culture and professional attitudes.^27^ The strategy on PHC professional nurses attending Wellness clinics was perceived to be important by some participants in order to promote PHC professional nurses’ well-being. Work-related stress, resulting from stressors such as high workloads as well as other issues like a lack of resources, has been found to be associated with poor job satisfaction.^28^ Stress, being overworked and lack of welfare facilities could decrease nurses’ satisfaction and the quality of healthcare provision.^29^

Improvements in how some PHC professional nurses performed their duties and engaged with each other, as observed in the manner they communicated with each other, were perceived as the results of the strategies that were implemented in some PHC clinics. The term ‘performance’ is used to focus attention on the total behaviour of a person, including his or her organisation, the use of specialised knowledge, his or her attitude acquired through training, as well as organisation and integration of practice. In this sense, performance-related behaviour is regarded to be directly associated with job tasks and the need to accomplish or achieve job objectives.^24^

Strategies that address essential functions of PHC professional nurses were perceived by the participants as vital to optimise and enhance effective caring for patients by PHC professional nurses. ‘Back to basics – Batho Pele Principles and Patients’ Rights Charter’ were perceived by the participants as essential in PHC professional nurses’ effective caring of patients, and to remind them of the various policies applicable to the workplace to treat patients with respect and dignity. Nursing seeks to ensure basic human care and decency, whilst the basic principles of human care suggest that patients should not be harmed, judged or degraded during a hospital stay.^30^ The strategy on reinforcement of nursing ethics was perceived by the participants as a means to help PHC professional nurses to abide by professional ethics at all times. Ethics is concerned with doing good and avoiding harm. Nurses are in a position to take decisions that affect the lives of patients, and their feelings about what is right and what is wrong will affect these decisions.^31^ Nurses who demonstrate a professional work ethic deliver a higher quality of care.^30^

The strategies that address the challenges faced by PHC professional nurses were perceived by the participants to assist them in dealing with issues such as absenteeism. Absenteeism is said to signify the intentional absence of an employee from work without any explanation and without authorisation and has a negative effect in terms of the quality of care rendered to patients. Unexcused absences lower productivity, result in low morale and are an added stress for other employees.^32^ As one of the barriers to effective caring for patients, absenteeism also affects organisations in terms of the quality of services provided and the loss of income.^33^

Participants perceived that strategies that facilitate treating patients equally without any discrimination will help them improve in managing patients from foreign countries. An aim when providing care in healthcare facilities is to achieve equal healthcare regardless of the patient’s gender, age or ethnicity.^34^ Rights-based reasoning refers to the belief that there are some things that are a person’s just due. This implies that all people, including foreign patients, have the right to fair and impartial treatment.^32^

Feedback from the study participants revealed that they would appreciate it if management can maintain the strategy that deals with addressing the shortage of PHC professional nurses in the PHC clinics, as this was seen as a factor compromising effective caring for patients. It can be mentioned that although many nurses are needed for the overburdened healthcare system, the shortage of nurses continues to widen with increasing demand without a commensurable increase in the supply of trained nurses.^35^ Staff shortages^24^ make it very difficult to provide a high standard of care.

A need to present the strategies to senior management by the researcher was identified by participants. This was perceived as a way that will motivate management to provide basic equipment in the PHC clinics. Without resources and support to meet workload demands, nurses grow dissatisfied and become emotionally exhausted.^36^ Some of the gaps such as resource constraints are identified as barriers to the implementation and sustainability of district health services.^37^

Based on the perceptions of the strategy that deals with managing the poor relationships between PHC professional nurses and patients, participants motivate for the strategy to be maintained in order to address the poor nurse–patient relationships and help maintain the positive relationships. In caring for patients, nurses attach great importance to effective communication, which is associated with effective listening, understanding and showing sensitivity towards patients.^38^ In caring for patients, nursing care^39^ is guided by a value base focusing on promoting dignity and health by means of caring relationships.

### Strengths and limitation of the study

The use of qualitative, in-depth, individual phenomenological interviews as the methodology to collect data serves as a strength of this study. Purposive sampling also enabled the researcher to select information-rich participants for the study. The limitations of this study refer to its inclusion of only two PHC clinics in Ekurhuleni, Gauteng province, with the view that these clinics might not be representative of all PHC clinics in Ekurhuleni.

### Recommendations

Based on the findings of the exploration and description of the PHC professional nurses’ and PHC nurse managers’ perceptions of the developed strategies in this study, it is recommended that the developed strategies should be regarded as a fundamental value to improve the culture of caring and to change the culture of how the PHC professional nurses work. This will ensure that respect, dignity and better experiences for both PHC professional nurses and patients are embedded in the nursing settings. To facilitate effective caring for patients by PHC professional nurses in public health clinics in Ekurhuleni, it will be beneficial to maintain the developed strategies to assist in resolving the identified problems. Because of the limited research studies that focus on exploring and describing perceptions of strategies to facilitate effective caring for patients by PHC professional nurses in PHC clinics, future studies that will focus on this aspect are recommended in order to add to the framework that guides nursing practice. The researchers also recommend further research to deepen the knowledge obtained by exploring the effects of the proposed strategies after the strategies are implemented in all PHC clinics in Ekurhuleni.

## Conclusion

The study explored and described PHC professional nurses’ and PHC nurse managers’ perceptions of the developed strategies to facilitate effective caring for patients in PHC clinics and three themes were identified: Theme 1, perceptions of strategies that focused on improving the healthcare system; Theme 2, perceptions of strategies that address essential functions of PHC professional nurses; and Theme 3, perceptions of strategies that address PHC professional nurses’ challenges. Strategies that focused on improving the healthcare system were perceived to have enabled strengthening of working relationships between PHC professional nurses and the PHC nurse manager. The strategies were also perceived to have brought some improvement in how PHC professional nurses performed their duties and how they communicated and engaged with each other. Strategies that address essential functions of PHC professional nurses were perceived to have enabled PHC professional nurses to correct their bad attitudes and to abide by professional ethics at all times. The participants perceived the strategies that address PHC professional nurses’ challenges to have brought a positive effect and improvement on the use of relevant policies of the institution, improved nurse–patient relationships and treating of patients from other countries with respect and dignity. Based on the findings of this study, it is recommended that the developed strategies and their implementation should be used as a supportive measure that can enable effective caring for patients in PHC clinics.

## Data Availability

Data sharing is not applicable to this article as no new data were created or analysed in this study.
